# Interspecific differences in oxidative DNA damage after hydrogen peroxide exposure of sea urchin coelomocytes

**DOI:** 10.1093/mutage/geac018

**Published:** 2022-09-20

**Authors:** Fengjia Liu, Kim S Last, Theodore B Henry, Helena C Reinardy

**Affiliations:** The Scottish Association for Marine Science, Oban, United Kingdom; The Scottish Association for Marine Science, Oban, United Kingdom; Institute of Earth and Life Sciences, School of Energy, Geoscience, Infrastructure and Society, Heriot-Watt University, Edinburgh, United Kingdom; Center for Environmental Biotechnology, The University of Tennessee, Knoxville, Knoxville, TN, United States; The Scottish Association for Marine Science, Oban, United Kingdom; Department of Arctic Technology, The University Centre in Svalbard, Longyearbyen, Norway

**Keywords:** DNA strand breaks, DNA damage, environmental effects, environmental toxicology, genotoxic effects in different organisms, genotoxicity

## Abstract

Interspecific comparison of DNA damage can provide information on the relative vulnerability of marine organisms to toxicants that induce oxidative genotoxicity. Hydrogen peroxide (H_2_O_2_) is an oxidative toxicant that causes DNA strand breaks and nucleotide oxidation and is used in multiple industries including Atlantic salmon aquaculture to treat infestations of ectoparasitic sea lice. H_2_O_2_ (up to 100 mM) can be released into the water after sea lice treatment, with potential consequences of exposure in nontarget marine organisms. The objective of the current study was to measure and compare differences in levels of H_2_O_2_-induced oxidative DNA damage in coelomocytes from Scottish sea urchins *Echinus esculentus*, *Paracentrotus lividus*, and *Psammechinus miliaris*. Coelomocytes were exposed to H_2_O_2_ (0–50 mM) for 10 min, cell concentration and viability were quantified, and DNA damage was measured by the fast micromethod, an alkaline unwinding DNA method, and the modified fast micromethod with nucleotide-specific enzymes. Cell viability was >92% in all exposures and did not differ from controls. *Psammechinus miliaris* coelomocytes had the highest oxidative DNA damage with 0.07 ± 0.01, 0.08 ± 0.01, and 0.07 ± 0.01 strand scission factors (mean ± SD) after incubation with phosphate-buffered saline, formamidopyrimidine-DNA glycosylase, and endonuclease-III, respectively, at 50 mM H_2_O_2_. Exposures to 0.5 mM H_2_O_2_ (100-fold dilution from recommended lice treatment concentration) induced oxidative DNA damage in all three species of sea urchins, suggesting interspecific differences in vulnerabilities to DNA damage and/or DNA repair mechanisms. Understanding impacts of environmental genotoxicants requires understanding species-specific susceptibilities to DNA damage, which can impact long-term stability in sea urchin populations in proximity to aquaculture farms.

## Introduction

The use of hydrogen peroxide (H_2_O_2_) as an antiparasitic treatment in Atlantic salmon aquaculture leads to dispersion into the marine environment and unintended exposure of surrounding marine organisms. The recommended treatment concentration and duration are 1.2–1.8 g/l (35–53 mM) H_2_O_2_ for 20 min [1], which induces a mechanical paralysis and detachment of the sea louse from the fish [2]. H_2_O_2_ is estimated to degrade into oxygen and water rapidly in just over 7 days [3], and is therefore considered to be the most environmentally friendly chemical option for sea lice treatment [4]. Although H_2_O_2_ is expected to dilute after release, hydrodynamic modelling has found that concentrations of 100–1000 times dilutions (530–53 µM) can persist over one kilometre from the release site, with speed and extent of dilution dependent on local weather and current conditions [5, 6]. H_2_O_2_ is denser than seawater and will sink to the seabed [7, 8]. The plume behaviour of H_2_O_2_ after a treatment event is therefore complex and dynamic, and both pelagic and benthic organisms are vulnerable to be exposed to elevated levels of H_2_O_2_ for hours or even days [8].

DNA damage can occur by exposure to environmental toxicants and lead to genome instability. Oxidative substances such as H_2_O_2_ that produce reactive oxygen species can cause oxidative DNA damage, including double- and single-stranded DNA breaks [4, 9–14]. Double- and single-stranded DNA breaks are the most harmful forms of DNA damage [13]. Unrepaired or misrepaired DNA strand breaks can lead to cell death, chromosome translocation, and genomic instability [15]. DNA strand breaks are often caused by oxygen radicals by inhibition in the progression of RNA polymerase [16]. Single-strand breaks can also deteriorate into the more lethal double-stranded DNA breaks by replication forks collapsing at sites of the break during replication [17]. In addition to DNA strand breaks, H_2_O_2_ can directly oxidize purine and pyrimidine deoxyribonucleotides where the former are more susceptible to oxidation than the latter as guanine has the lowest oxidation potential of all four nucleobases [18]. Unrepaired oxidized purine and pyrimidine lesions can lead to mutagenesis, cytostasis, and cytotoxicity [19].

Marine organisms including sea urchins share the coastal environment with anthropogenic activities; therefore, they can be useful models for assessment of genotoxic impacts from discharge of contaminants into the surrounding ecosystem. Sea urchins are found all over the world with extensive nearshore salmon aquaculture in Scotland, Chile, and Norway. Several species of sea urchins are found in abundance in proximity of salmon farms on the west coast of Scotland, with access to salmon cage waste [20, 21]. With the release of H_2_O_2_ from salmon farms, slower moving sea urchins can be directly impacted by waste plumes, making them a useful bioindicator for sea lice treatment effects. Those inhabiting coastal areas can have ecological roles influenced by human activities and previous studies have used them as bioindicators for local pollutants [22–24]. They are pivotal organisms in the maintenance of ecological balance in the marine ecosystem and are found in all marine environments [25, 26]. European lobsters at pelagic larvae stage [27], deep-water shrimp [8], zooplankton [28], and krill [29] have all been observed to suffer adverse behavioural and genotoxic effects, when exposed to 10–1000 times dilutions (53–5.3 mM) of the recommended sea lice treatment, and sublethal behavioural changes observed at 100–200 times dilutions (265–530 µM). It is unknown how sea urchin coelomocytes will respond to realistic environmental concentrations of H_2_O_2_, however their role as a benthic bioindicator species [30], along with their ease of collection, maintenance, husbandry, cell extraction, transparency and large quantities of embryos, and larvae, make them an ideal model for investigating H_2_O_2_ impacts.

There are several methods for DNA damage detection and measurements. The fast micromethod detects single- and double-stranded DNA breaks, and alkali labile sites by means of alkaline unwinding, following the same first principles of alkaline DNA unwinding in the comet assay, but with more scope for other cell systems, sensitivity, larger sample sets, and more rapid quantification [13, 31, 32]. Studies have used the fast micromethod to detect DNA damage in sea urchin coelomocytes after short exposures to H_2_O_2_ [12, 33, 34], and the current study looks to further investigate oxidized purine and pyrimidine nucleotides caused by H_2_O_2_ in addition to strand breaks. Previously, the comet assay was modified to incorporate enzymes into the protocol for lesion-specific DNA damage detection [35]. Formamidopyrimidine-DNA glycosylase (FPG) and endonuclease-III (Endo III) are bacterial repair enzymes used in the modified comet assay for the measurement of oxidized purines and pyrimidines and they have been identified as efficient biomarkers for detecting oxidative DNA damage [36, 37]. FPG and Endo III work by recognizing and cleaving oxidized purine and pyrimidine nucleotides, respectively, converting the oxidative damage into single strand breaks [38]. Compared with the comet assay, the fast micromethod is a more rapid method for determining DNA damage suitable for large quantity of samples in ecotoxicological studies [31]. Whereas the comet assay separates and spreads unwound DNA by electrophoresis, the fast micromethod incorporates PicoGreen which preferentially binds to double-stranded DNA and is kinetically released during the alkaline process of unwinding, allowing for quantification of the rate of release (reduction in fluorescence) correlated with initial levels of DNA strand breaks.

The aim of the current study was to measure and compare levels of oxidative DNA damage in coelomocytes of cold-water sea urchin species *Echinus esculentus*, *Paracentrotus lividus*, and *Psammechinus miliaris* from the west coast of Scotland and within the vicinity of Atlantic salmon aquaculture. The objectives were: (i) develop enzyme modified fast micromethod for investigating lesion-specific DNA damage in coelomocytes; (ii) identify and differentiate oxidative DNA damage susceptibility between the three sea urchins species; (iii) differentiate and quantify H_2_O_2_-induced DNA strand breaks, oxidized purines, and oxidized pyrimidines; and (iv) interpret results in the context of interspecific differences in size, coelomocyte characteristics, and within the context of environmental exposure to H_2_O_2_ antiparasitic treatments in coastal Atlantic salmon aquaculture.

## Materials and methods

### Animal maintenance

Adult stocks of *E. esculentus*, *P. lividus*, and *P. miliaris* were maintained at the Scottish Association for Marine Science (SAMS) aquarium in a flow-through system with filtered natural seawater, ambient temperature, and photoperiod, and were fed *ad libitum* with macroalgae. Sea urchins used in the current study were selected from adult stocks kept in the aquarium for up to 9 years. Animals were maintained and handled in accordance with UK animal welfare regulations. Routine aquarium husbandry and experimental protocols received ethical approval from University of Highlands and Islands research ethics committee and animal welfare and ethics committee (application ID: 503). Each sea urchin was measured to determine test diameter and height before coelomic fluid extraction, and in total, 45 of *E. esculentus*, 31 of *P. lividus*, and 12 of *P. miliaris* were used in the current study.

### Coelomocyte extraction and H_2_O_2_ exposure

Coelomocytes were collected by sublethal extraction of coelomic fluid (0.5–1 ml) with a syringe and an 18- or 21-gauge needle inserted at a 45° angle through the peristomial membrane surrounding the Aristotle’s lantern. Syringes were prefilled with ice-cold calcium/magnesium-free seawater (460 mM NaCl, 10 mM KCl, 7 mM Na_2_SO_4_, 2.4 mM NaHCO_3_) containing 30 mM ethylenediaminetetraacetic acid (EDTA) anticoagulant [pH 7.4, 1:1 (vol:vol), with coelomic fluid] to prevent cells clumping [39]. Cell concentration and differential cell counts (red spherule or clear cells) were determined with a Neubauer haemocytometer. Cells were diluted to 2.78 M/ml with 30 mM EDTA anticoagulant. In total, coelomocyte concentrations from *n* = 45 *E. esculentus*, *n* = 31 *P. lividus*, and *n* = 12 *P. miliaris* were recorded, coelomocytes from *n* = 8 *E. esculentus*, *n* = 4 *P. lividus*, and *n* = 4 *P. miliaris* were used for the fast micromethod, and coelomocytes from *n* = 6 *E. esculentus*, *n* = 6 *P. lividus*, and *n* = 6 *P. miliaris* were used for the modified fast micromethod.

Coelomocytes were exposed to H_2_O_2_ [35% stabilized H_2_O_2_ (w/w), VWR international Ltd, Leicestershire, UK] at 1:10 ratio to reach final H_2_O_2_ concentrations of 0.5–50 mM, PBS at 1:10 ratio was added to control coelomocytes. Samples were exposed for 10 min on ice and in the dark, same as acute H_2_O_2_ exposures in previous research on DNA damage in sea urchins [33]. After exposure, subsamples were placed on a Neubauer haemocytometer with 0.5% trypan blue at 1:1 ratio, and cell viabilities were assessed by counting of stained blue dead cells and clear live cells.

### DNA damage detection

After exposure to H_2_O_2_, coelomocytes were aliquoted into quadruplicate wells (96-well black-walled microplates, Greiner Bio-One Ltd) with cell concentration of ~50 000 cells per well, consistent with previously published method from Reinardy and Bodnar [12]. Lysis solution (9 M urea, 0.1% SDS, 0.2 M EDTA, pH 10) with PicoGreen (1:50, Fisher Scientific, Leicestershire, UK) was added to each well, plate planks contained PBS without coelomocytes, and lysed in the dark on ice for 40 min. To initiate DNA unwinding, 200 µl unwinding solution (1 M NaOH, 20 mM EDTA, pH 13) was added to each well, fluorescence was immediately read by kinetic mode every 5 min for 20 min (POLARstar Omega, excitation wavelength at 480 nm and emission wavelength at 520 nm, BMG LABTECH Ltd, Bucks, UK).

The fast micromethod was modified to include enzyme-specific incubations for differentiation of oxidized purines and pyrimidines, following the principle established for the modified comet assay [36, 40]. Following separate H_2_O_2_ exposure experiments, lysis solution was added to the coelomocytes in 1.5 ml microcentrifuge tubes at 1:1 (vol:vol) ratio and cells were lysed in the dark and on ice for 40 min. After lysis, each sample was split into three subsamples and were either incubated with PBS (no enzyme set), FPG (0.04 units), or Endo III (0.05 units) (New England Biolabs, Herts, UK). FPG and Endo III were diluted at 1:1000 with Milli-Q water. Enzymes or PBS control were added to the coelomocytes, followed by incubation in a thermo cycler (MJ Research, Inc., PTC-100TM Programmable Thermo Controller) for 30 min at 37°C and 40 min deactivation step at 4°C [40, 41]. After incubation, cells (approx. 50 000 cells/well) were loaded into quadruplicate wells. PicoGreen, diluted with PBS with ratio of 1:10, was added to each reaction for the fluorescence binding of double-stranded DNA and allowed to sit on ice for 10 min. DNA unwinding was initiated and fluorescence was read same as the fast micromethod.

### Data analysis

Total cell concentration, red cell percentage, cell viability, test diameter, and height datasets in *E. esculentus*, *P. lividus*, and *P. miliaris* were tested with one variable analysis Shapiro–Wilk test for normal distribution. Total cell concentrations and test diameters for all species were normally distributed. Red cell percentages were normally distributed for *P. miliaris* but not normally distributed for *E. esculentus* and *P. lividus*. Test heights were normally distributed for *P. lividus* and *P. miliaris*, but not normally distributed for *E. esculentus*. Cell viabilities were not normally distributed for all species. Nonparametric Kruskal–Wallis one-way analysis of variance on ranks (ANOVA on Ranks) followed by a *post hoc* Dunn’s method was used to compare differences between species for datasets that were not normally distributed, and one-way ANOVA was used to compare differences between species for datasets that were normally distributed.

DNA damage measured by fast micromethod was calculated by the strand scission factor (SSF) equation: SSF=log(%dsDNAsample/%dsDNAcontrol)×(−1) [12, 31, 33]. Fast micromethod data were normally distributed and tested with ANOVA for concentration-dependent increases of DNA damage (SSF). Concentration-dependent DNA damages were also modelled by three-parameter logistic regression $f=a/(1+exp{\left(-(x-x_{0})\right)}^{b})$) for the fast micromethod [12]. Modified fast micromethod data were normally distributed and statistically tested with general linear model (GLM) for concentration-dependent increases of SSF, and ANOVA to determine the differences between no enzyme, FPG, and Endo III sets of data at different concentrations of H_2_O_2_. Three-parameter logistic regression was also performed on no enzyme, FPG, and Endo III datasets separately for statistical testing of concentration-dependent DNA damage responses. All statistically analyses were performed in SigmaPlot 14.0 (Systat Software, Inc.) or Statgraphics 19 (Statgraphics Technologies, Inc., VA, USA).

## Results

Out of the three species of sea urchins, *P. miliaris* was the smallest (*P* < .05, ANOVA or Kruskal–Wallis) with diameter of 39 ± 3.5 (means ± SD, *n* = 12) mm and height of 21 ± 3.0 (means ± SD, *n* = 12) mm (Table 1). No significant differences in size were found between *E. esculentus*, diameter of 46 ± 4.6 mm and height of 24 ± 4.2 mm, and *P. lividus*, diameter of 46 ± 9.4 mm and height of 24 ± 5.1 mm (*P* > .05, ANOVA or Kruskal–Wallis). All three species had total coelomocytes concentrations of 10.24–13.02 × 10^6^ cells/ml and red spherule cells percentage of 4.2%–9.5% (means ± SD) (Table 1). No significant differences in total cell concentrations were found (*P* > .05, ANOVA), but *E. esculentus* had a significantly lower percentage of red cells of 4.2 ± 4.3% compared with *P. lividus* and *P. miliaris* (*P* < .05, ANOVA). Red cell percentages of *P. lividus*, 6.9 ± 3.8%, and *P. miliaris*, 9.5 ± 4.9%, were not significantly different (*P* > .05, Kruskal–Wallis). Cell viabilities, as recorded in fast micromethod and modified fast micromethod in coelomocytes in all species exposed to 50 mM H_2_O_2_ was over 92% (Table 1) with no significant difference in cell viabilities between species (*P* > .05, Kruskal–Wallis).

**Table 1.** Sea urchin sizes and coelomocytes concentrations.

Sea urchin species	*n*	Test diameter (mm)	Test height (mm)	Red spherule cells (% of total coelomocyte concentration)	Total coelomocyte concentration (×10^6^ cells/ml)	Cell viability at the highest H_2_O_2_ concentration (%)
*E. esculentus*	45	46 ± 4.6	24 ± 4.2	4.2 ± 4.3*	11.8 ± 4.2	96.6 ± 2.6
*P. lividus*	31	46 ± 9.4	24 ± 5.1	6.9 ± 3.8	13.02 ± 5.8	97.3 ± 1.0
*P. miliaris*	12	39 ± 3.5*	21 ± 3.0*	9.5 ± 4.9	10.24 ± 2.7	98.2 ± 1.0

*E. esculentus* (*n* = 45), *P. lividus* (*n =* 31), and *P. miliaris* (*n =* 12) test diameter and height shown in mean ± SD mm. Total cell concentration shown in mean ± SD × 10^6^ cells/ml and red spherule cells in mean ± SD % of total coelomocyte concentration. Cell viability assessed with 0.5% trypan blue on coelomocytes exposed to 50 mM H_2_O_2_ and shown in mean ± SD %.

*Significant differences in test diameter and height, and red spherule cells % (*P* < .05, ANOVA or Kruskal–Wallis).

**Figure 1.**
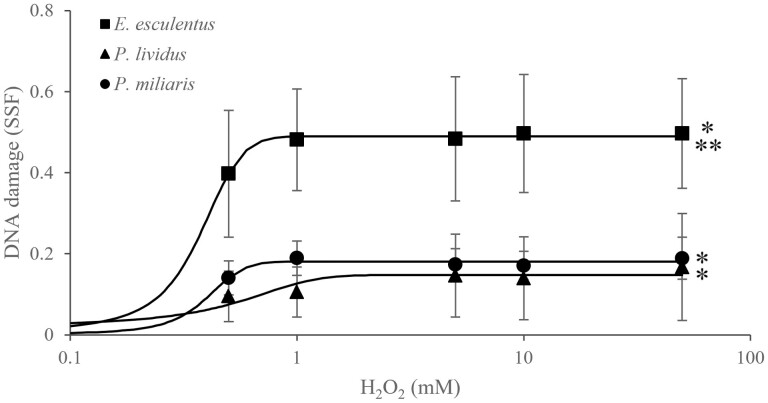
H_2_O_2_ concentration-dependent DNA damage (SSF, fast micromethod) of coelomocytes in three species of sea urchins, *E. esculentus* (■, *n* = 8), *P. lividus* (▲, *n* = 4), and *P. miliaris* (●, *n* = 4). Data were modelled with three-parameter logistic regression and data points are means ± SD (*n* = 4–8).). *Significant concentration-dependent increase in DNA damage (*P* < .05, ANOVA). **Significant species difference in overall level of SSF (*P* < .05, ANOVA).

*Echinus esculentus* (*n* = 8), *P. lividus* (*n* = 4), and *P. miliaris* (*n* = 4) coelomocytes DNA damage (SSF) increased after exposure to 0–50 mM H_2_O_2_ (Fig. 1). At 50 mM of H_2_O_2_, *P. lividus* and *P. miliaris* had a maximum mean level of DNA damage of 0.17 ± 0.13 and 0.19 ± 0.05 SSFs, respectively, whereas *E. esculentus* had the highest DNA damage at 0.50 ± 0.14 SSF (Fig. 1). *Echinus esculentus* also had significantly the highest SSFs of all three species with 0.40 ± 0.16, 0.48 ± 0.13, 0.48 ± 0.15, 0.50 ± 0.14, and 0.50 ± 0.14 SSFs after exposure to 0.5, 1, 5, 10, and 50 mM of H_2_O_2_ (*P* < .05, ANOVA), respectively, while no significant difference in SSFs was found between *P. lividus* and *P. miliaris* (*P* > .05, ANOVA). Acute exposures to 0.5–50 mM H_2_O_2_ significantly induced DNA damage in *E. esculentus* and *P. miliaris* (*P* < .05, ANOVA), and 5–50 mM of H_2_O_2_ significantly induced DNA damage in *P. lividus* (*P* < .05, ANOVA).

**Figure 2.**
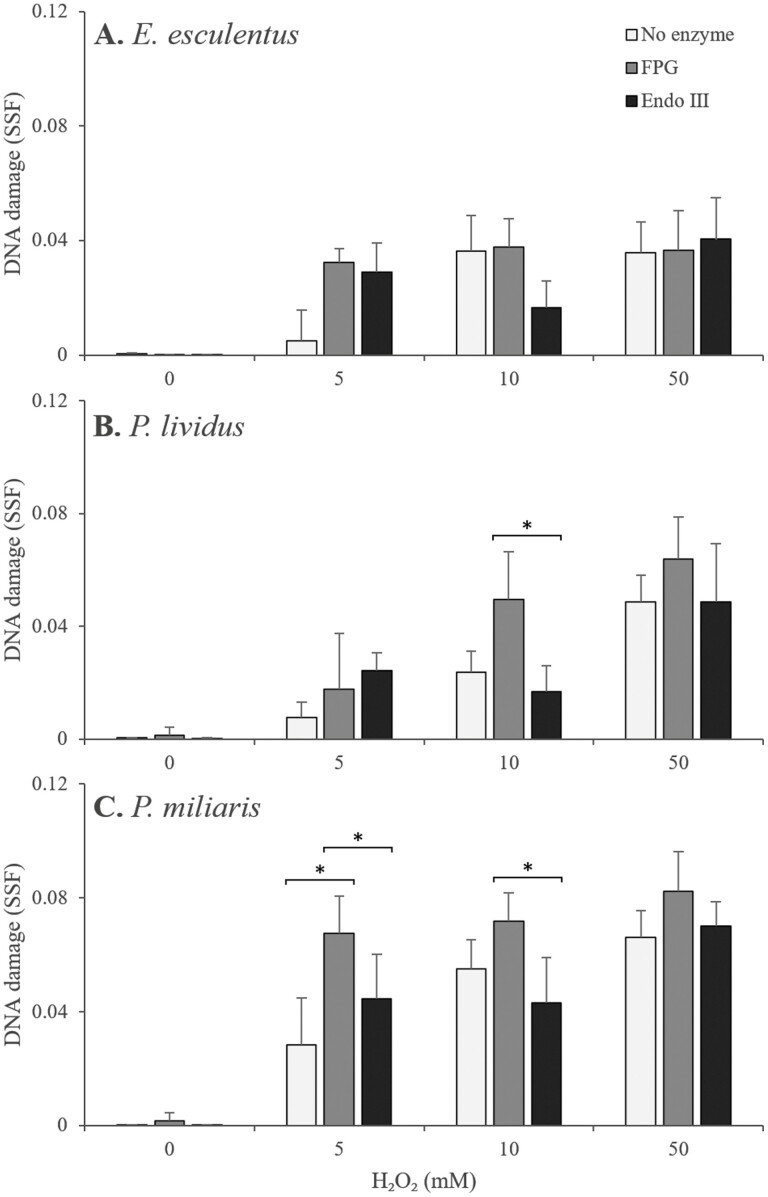
DNA damage (SSF) in *E. esculentus* (*n* = 6, A), *P. lividus* (*n* = 6, B), and *P. miliaris* (*n* = 6, C) coelomocytes exposed to H₂O₂. Fast micromethod modified to include either no enzyme (white bars), incubation with FPG (grey bars), or incubated with Endo III (black bars). Data are means ± SD, *Significant difference in SSF between no enzyme, FPG, and Endo III (*P*﻿ < .05, GLM). DNA damage SSF was significantly affected by H₂O₂ concentrations for all treatments (*P*﻿ < .05, GLM).

Coelomocytes exposed to H_2_O_2_ (0–50 mM) from *E. esculentus* (*n* = 6), *P. lividus* (*n* = 6), and *P. miliaris* (*n* = 6) then incubated with no enzyme, FPG, and Endo III showed significant H_2_O_2_-induced oxidative DNA damage (*P* < .05, GLM, Fig. 2). Concentration-dependent DNA damage responses fitted three-parameter logistic regression models significantly (*P* < .05, logistic regression) for no enzyme, FPG, and Endo III incubated coelomocytes from all species of sea urchin. Significant differences in enzyme treatments were found in *P. lividus* at 10 mM H_2_O_2_ between FPG and Endo III (*P* < .05, GLM), *P. miliaris* at 5 mM H_2_O_2_ between no enzyme, FPG, and Endo III (*P* < .05, GLM), and *P. miliaris* at 10 mM H_2_O_2_ between FPG and Endo III (*P* < .05, GLM). In *E. esculentus* at 5 mM H_2_O_2_, coelomocytes with FPG and Endo III show higher DNA damage compared with coelomocytes with no enzymes. At 10 mM H_2_O_2_, coelomocytes with no enzyme and FPG show similar SSF, whereas coelomocytes with Endo III had lower SSF. At 50 mM H_2_O_2_, all three sample sets showed similar SSF. DNA damage reached a plateau at 10 and 50 mM H_2_O_2_ (Fig. 2A). No significant DNA damage was observed between coelomocytes incubated with no enzyme, FPG, and Endo III for all concentrations of H_2_O_2_ in *E. esculentus* (*P* > .05, GLM, Fig. 2A). *Paracentrotus lividus* coelomocytes incubated with no enzyme showed lower but nonsignificant SSF in 5 mM H_2_O_2_ compared with samples with FPG and Endo III at (*P* > .05, GLM, Fig. 2B). Coelomocytes incubated with Endo III had significantly lower SSFs (*P* < .05, GLM) compared with coelomocytes incubated with no enzymes and FPG when treated with 10 mM H_2_O_2_ (Fig. 2B). At 50 mM, coelomocytes incubated with FPG showed the highest SSF compared with no enzyme and FPG, but it was not significant (*P* > .05, GLM, Fig. 2B). *Paracentrotus lividus* coelomocytes for all enzyme treatments also showed highest SSFs at 50 mM, but not significantly different than at 5 or 10 mM (*P* > .05, GLM, Fig. 2B). *Psammechinus miliaris* showed the highest overall damage levels of SSFs of the three species of sea urchins with concentration-dependent DNA damage (Fig. 2C). Coelomocytes incubated with no enzyme had significantly lower SSF (*P* < .05, GLM) at 5 mM H_2_O_2_ and coelomocytes incubated with Endo III had significantly lower SSF (*P* < .05, GLM) at 10 mM H_2_O_2_ (Fig. 2C). *Psammechinus miliaris* coelomocytes incubated with FPG had the highest DNA damage at every treatment concentration of H_2_O_2_, similar to *E. esculentus* at 5 and 10 mM H_2_O_2_ and *P. lividus* at 10 and 50 mM H_2_O_2_. At both 5 and 10 mM of H_2_O_2_, significant differences in DNA damage were observed between FPG and Endo III, and between no enzyme and FPG at 5 mM H_2_O_2_ (*P* < .05, GLM, Fig. 2C).

## Discussion

Total cell concentration and percentage of red cells recorded in the current study indicated large variations between individual sea urchins and different species having similar ranges in concentrations. Sea urchins have a complex innate immune system where coelomocytes play a key immunological role and the current study differentiated coelomocytes into red spherule cells and clear cells (white spherule cell, phagocytes, and vibratile cells) [39, 42, 43]. Cell concentrations reported in the current study were higher than previous studies. Both *P. lividus* and *P. miliaris* reported to have 6–8 × 10^6^ cells/ml, whereas the current study reports the two species with averages of 13.02 × 10^6^ cells/ml and 10.24 × 10^6^ cells/ml, respectively [23, 44, 45]. In addition, previous research suggests that sex and proximity to spawning season contributes to the amount of cells present as female and prespawning sea urchins had higher coelomocyte concentrations at 8.2 ± 1.2 × 10^6^ cells/ml and 7.3 ± 1.7 × 10^6^ cells/ml for females and males prespawning, compared with 7.1 ± 2.1 × 10^5^ cells/ml and 6.8 ± 2.7 × 10^5^ cells/ml for females and males postspawning [44]. The current study extracted the coelomocytes from prespawning sea urchins in the winter months and did not distinguish the sexes of sea urchins, which could be contributing to the large variations in coelomocytes concentrations. *Psammechinus miliaris* had the highest average percentage (9.5%) of red cells, which is consistent with previous studies [45], compared with *E. esculentus* (4.2%) and *P. lividus* (6.9%). Coelomocytes function similar to vertebrate blood cells as the main line of defence against wounding and infections by opsonization, coagulation, encapsulation, phagocytosis, clearing foreign particles, and oxygen transport [39, 46–48]. Red spherule cells contain echinochrome A, which has antibacterial and antifungal activities and have been used as a biomarker for heat stress [49, 50]. Sea urchin coelomocytes are known to vary the proportions of red to white coelomocytes between species and individuals according to size and physiological conditions, and the number of red spherule cells can increase when exposed to polluted seawater or injuries [51]. The three species used in the current study had different average sizes with *P. miliaris* the smallest, which is consistent with previous records [21, 52, 53]. Susceptibility to environmental stress and their natural small sizes or temperature tolerance could also contribute to the relatively high red cell percentage in *P. miliaris*. *Psammechinus miliaris* are distributed across the North Sea, far into Scandinavia, and are tolerant to low temperatures down to 0°C [21]. The water temperature during the time of sampling for the current study was >10°C, possibly warmer than what *P. miliaris* is most comfortable in. *Echinus esculentus* has the lowest red cell percentage which may indicate a reduced capacity to respond to stress, or an overall unstressed state.

For accurate estimation of DNA damage in coelomocytes, cell viability was first assessed to confirm the presence of live coelomocytes. Sea urchin coelomocytes are suggested to be relatively tolerant to high concentrations of genotoxicants and have similar DNA repair mechanisms as vertebrates with a range of repair pathways, suggesting a robust DNA damage response system [12, 54–56]. Coelomocytes in the current study underwent sublethal H_2_O_2_ exposures of 0.5–50 mM, equivalent to 1- to 100-fold dilution from recommended sea lice treatment regime within an Atlantic salmon farm [1]. The cell viabilities of coelomocytes exposed to H_2_O_2_ concentrations of 0 and 50 mM were assessed after every exposure and coelomocytes from every sea urchin had cell viability of >92%. No significant differences were observed in cell viabilities between species of sea urchins. Coelomocytes acutely exposed to 10 mM H_2_O_2_ had an average of 99% viability and modelled LC_50_ at 120.1 mM, compared with 21% average viability and modelled LC_50_ at 0.6 mM for sea urchin larvae [12]. Human cell systems have shown 5%–60% cell viability at 10 mM and <5% at 400 µM H_2_O_2_ exposure for 24 h [57, 58]. *Poeciliopsis* fish hepatoma cells showed <20%, <20%, and <10% cell viabilities after exposure to 0.1, 1, and 10 mM of H_2_O_2_ for 24 h [59]. Little is known on the range of acute responses to H_2_O_2_ across marine invertebrate groups and compared with sea urchin cell viabilities. With the minimum of 92% cell viability at a high H_2_O_2_ concentration of 50 mM, sea urchin coelomocytes are relatively tolerant to genotoxicity, and the results confirm that exposures were sublethal and DNA damage assessments were conducted in live coelomocytes.

The fast micromethod detected significant DNA damage in sea urchin coelomocytes exposed to 0.5–50 mM H_2_O_2_. DNA damage reached the maximum levels at 1 mM H_2_O_2_ for all species, beyond which, there was no increase in damage. Previous research showed highest DNA damage from H_2_O_2_ at ≥1 mM in both adult coelomocytes and larvae reaching maximum SSFs at 1 mM H_2_O_2_ in the subtropical sea urchin *Lytechinus variegatus* [12]. Reinardy and Bodnar [12] followed the fast micromethod SSF, providing comparable data to the present study, and reported SSFs of the same range and magnitude, and maximum 0.5 SSF, similar range was found in *E. esculentus* from the current study, but higher than what was detected in *P. lividus* and *P. miliaris*. This may be explained by interspecific differences, geographical ranges and environments, methodological differences used i.e. different fluorescence spectrometers, or differences in the pH of alkaline unwinding solutions with pH 12.4 for *L. variegatus* DNA unwinding and pH 13 in the present study. A study using the fast micromethod in coelomocytes from multiple species of echinoderms reported levels of DNA damage ranging from 0.5 to 1.5 SSF across three difference species, and suggested longer-lived echinoderms may invest more in DNA damage tolerance compared with short-lived species [33]. Both Reinardy and Bodnar [12] and El-Bibany *et al.* [33] investigated sea urchin species from subtropical environment in Bermuda as opposed to the cold-water species from the current study, and they quantified levels of DNA damage up to 1.5 SSF, suggesting species from different geographical and thermal conditions may have differences in DNA damage susceptibility and capacity to respond to environmental genotoxicants.

H_2_O_2_-induced oxidative DNA damage was detected with the modified fast micromethod with FPG and Endo III incubated coelomocytes, indicating H_2_O_2_ caused DNA strand breaks in addition to direct oxidation of purines and pyrimidines. The modified fast micromethod was developed to detect oxidized DNA damage caused by H_2_O_2_ with added FPG and Endo III enzyme incubations. It was able to detect, quantify, and differentiate oxidized purine and pyrimidine nucleotides as well as DNA strand breaks in coelomocytes from different species of sea urchins and sensitive enough to differentiate between species and H_2_O_2_ concentrations. Compared with the modified comet assay, the modified fast micromethod is able to be performed more rapidly and work on multiple tissue and cell types, and large sample sizes, with the further potential to apply to DNA extracted from any species or tissue type. FPG incubated coelomocytes resulted in the highest SSF across all species, suggesting that H_2_O_2_ caused more oxidized purine nucleotides than oxidized pyrimidine nucleotides. Previous studies that used FPG and Endo III modified comet assay indicated and support purines being more susceptible to oxidation than pyrimidines [60, 61]. Coelomocytes where no significance was found between no enzyme and FPG incubations suggest the DNA damage caused by H_2_O_2_ mainly consisted of single-, double-stranded DNA breaks, and alkali labile sites. The current study provides strong evidence that H_2_O_2_ mainly causes strand breaks and more oxidized purines than pyrimidines. *Echinus esculentus* had the highest DNA damage detected by the fast micromethod suggesting it to be the most susceptible species in the current study. However, *P. miliaris* had the highest DNA damage in all enzyme treatments using the modified fast micromethod. This may be explained in that the modified fast micromethod takes a total of 4 h compared with 2.5 h for the unmodified fast micromethod. Previous research showed sea urchins to have robust DNA repair capabilities, with repair initiated and progressing within 1 h [12]. This suggests that the DNA damage detected by the modified fast micromethod was possibly not the maximum DNA damage caused by H_2_O_2_, but the net DNA damage after some early DNA repair processes have been initiated. DNA damage susceptibility can be considered as the sum of DNA protection, DNA damage, and biochemical responses to that damage (DNA repair, antioxidant activity), which can vary between cell types (e.g. differences in nuclear or mitochondrial DNA [62]), between species [33], and life strategies (e.g. longevity [63]). Therefore, time differences between the un- and modified fast micromethod could exacerbate the DNA damage response systems (repair and/or antioxidant activity), suggesting that *P. miliaris* may have slightly lower capacity for DNA damage response and higher overall DNA damage susceptibility than the other two species. These interspecific differences in initial vulnerability to DNA damage, and the capacity to initiate a robust and effective DNA repair response, warrants further investigation, and the modified fast micromethod could provide a rapid high-throughput method for multiple species and timepoints.

The current study concludes with further evidence of the potential harm that H_2_O_2_ can cause in benthic invertebrates in the marine environment around fish farms using H_2_O_2_ treatment against sea lice. The newly developed FPG and Endo III modified fast micromethod was able to detect and quantify oxidized bases, and differentiate between DNA strand breaks, oxidized purines, and oxidized pyrimidines. Data from the modified fast micromethod indicate that H_2_O_2_ causes oxidative DNA damage in sea urchin coelomocytes and that it induces predominately DNA strand breaks, and more oxidized purines than pyrimidines. The three sea urchin species from the same cold-water environment have different levels of H_2_O_2_-induced oxidative DNA damage, suggesting differences in DNA damage susceptibilities, or overall DNA damage response systems including capacity for DNA repair. Sea urchin test size, cell concentration, and red cell percentage showed large variations between individuals and provide comparisons between the temperate species important in coastal ecosystems hosting Atlantic salmon aquaculture industrial activity. Furthermore, the impact of H_2_O_2_ may be more extensive in other benthic and sessile marine organisms given that sea urchins have a very well-developed immune system with high resilience to DNA damage from environmental stressors [34]. Finally, data from the current study show genotoxic effects on sea urchin coelomocytes from environmentally relevant concentrations of H_2_O_2_ and indicate H_2_O_2_ releases from fish farms’ potential for genotoxic impact on nontarget marine organisms.

## Data Availability

The datasets generated for this study are available on request to the corresponding author.
